# Morphological, Spectroscopic and Thermal Analysis of Cellulose Nanocrystals Extracted from Waste Jute Fiber by Acid Hydrolysis

**DOI:** 10.3390/polym15061530

**Published:** 2023-03-20

**Authors:** Md. Sohel Rana, Md. Abdur Rahim, Md. Pervez Mosharraf, Md. Fazlul Karim Tipu, Jakir Ahmed Chowdhury, Mohammad Rashedul Haque, Shaila Kabir, Md. Shah Amran, Abu Asad Chowdhury

**Affiliations:** 1 Department of Pharmaceutical Chemistry, Faculty of Pharmacy, University of Dhaka, Dhaka 1000, Bangladesh; 2 Department of Pharmacy, Faculty of Pharmacy, University of Dhaka, Dhaka 1000, Bangladesh; 3 Department of Pharmaceutical Technology, Faculty of Pharmacy, University of Dhaka, Dhaka 1000, Bangladesh

**Keywords:** jute, cellulose nanocrystals, acid hydrolysis, particle size, thermal stability

## Abstract

Natural cellulose, a sustainable bioresource, is highly abundant in nature. Cellulosic materials, particularly those that explore and employ such materials for industrial use, have recently attracted significant global attention in the field of material science because of the unique properties of cellulose. The hydroxyl groups enable the formation of intra- and inter-molecular hydrogen bonding and the arrangement of cellulose chains in a highly ordered crystalline zone, with the remaining disordered structure referred to as an amorphous region. The crystalline areas of cellulose are well-known as cellulose nanocrystals (CNCs). In the present study, we extracted CNCs from pure cellulose isolated from waste jute fibers by sulfuric acid hydrolysis, followed by characterization. Pure cellulose was isolated from jute fibers by treating with sodium hydroxide (20% *w*/*w*) and anthraquinone (0.5%) solution at 170 °C for 2 h, followed by bleaching with chlorine dioxide and hydrogen peroxide solution. CNCs were isolated from pure cellulose by treating with different concentrations (58% to 62%) of sulfuric acid at different time intervals (20 min to 45 min). The FTIR study of the CNCs reveals no peak at 1738 cm^−1^, which confirms the absence of hemicellulose in the samples. The CNCs obtained after 45 min of acid hydrolysis are rod-shaped, having an average length of 800 ± 100 nm and width of 55 ± 10 nm, with a high crystallinity index (90%). Zeta potential significantly increased due to the attachment of SO_4_^2−^ ions on the surface of CNC from −1.0 mV to about −30 mV, with the increment of the reaction time from 20 min to 45 min, which proved the higher stability of CNC suspension. Crystallinity increased from 80% to 90% when the reaction time was increased from 20 to 45 min, respectively, while a crystallite size from 2.705 to 4.56 nm was obtained with an increment of the acid concentration. Acid hydrolysis enhanced crystallinity but attenuated the temperature corresponding to major decomposition (Tmax) at 260 °C and the beginning of degradation (Ti) at 200 °C due to the attachment of SO_4_^2−^ ions on the surface, which decreased the thermal stability of CNC. The second degradation at 360 °C indicated the stable crystal structure of CNC. The endothermic peak at 255 °C in the DTA study provided evidence of sulfated nanocrystal decomposition and the recrystallization of cellulose I to cellulose II, the most stable structure among the other four celluloses. The proposed easy-to-reproduce method can successfully and efficiently produce CNCs from waste jute fibers in a straightforward way.

## 1. Introduction

French scientist Anselme Payen discovered cellulose in 1838 after isolating it from plant tissue and figuring out its chemical composition [1]. Cellulose is a water-insoluble fibrous material and the most prevalent natural polymer on the planet, as well as an essential structural constituent of the cell wall of a variety of plants. In addition to plants, it is also found in a vast array of other organisms, including algae, fungi, bacteria, and even tunicates [2]. Cellulose is made of glucopyranose units through β-1,4-linkage with the formation of a high molecular weight, long-chain homopolymer, in which each monomer component is coiled at an angle of 180° concerning its neighbors [3,4]. Cellulose I, II, III_I_, III_II_, IV_I_, and IV_II_ are six interchangeable polymorphs whose constituents differ by source [5]. Native cellulose I is present in microorganisms, algae, higher plants, and tunicate [6,7]. Cellulose I conversion into cellulose II form is seen in mercerized cotton and regenerated cellulose. Cellulose III is developed by processing native cellulose with ethylamine or ammonia. Heating cellulose III_I_ or III_II_ in glycerol at 260 °C generates polymorphs IV_I_ and IV_II_ [3,8,9].

Cellulose nanocrystals (CNCs), also known as nano-whiskers, are rigid, rod-shaped, or spherical particles of 30 nm or less in width and a few hundred (100–1000) nm long, composed of cellulose chain fragments with an almost flawless crystalline arrangement [3,10]. Nanocrystals are more robust, biocompatible, sustainable, stiffer, renewable, optically transparent, nontoxic, lightweight, gas impermeable, biodegradable, modulus, and liquid crystalline than bulk cellulose [11,12]. These outstanding properties draw the attention of global researchers, consequently leading to vast application of CNC in numerous fields, such as the pharmaceutical industry, food industry, polymer industry, paper-making industry, catalysis, energy and electronic sector, environment sector, and so on [4,13,14,15,16].

In the very beginning, Nickerson and Habrle used acid hydrolysis to prepare CNCs, where Rnby discovered micro-cellulose suspension in 1951 [4,12]. According to the literature, CNCs are also isolated from various natural resources, specifically from agro-waste such as rice straw, corn husk, cotton linter, sugar palm fibers, oil palm fronds, cassava bagasse, doum leaves, pineapple leaves, sugarcane bagasse, coconut husk fibers, banana, mengkuang leaves, apple pomace, kenaf bast, bamboo, and date palm [12,17,18]. To synthesize CNCs and nanocellulose, different methods and processes were addressed by various researchers, such as hydrolysis with sulfuric acid [18], hydrochloric acid [19], phosphoric acid [20], formic acid [21], the mechanical method [22], the enzymatic method [23], treatment with ionic liquid [24], the oxidative method [25], ultrasonic-chemical mixed [26], and the ultrasonic method [27]. The performance, morphology, and properties of CNCs greatly depend on the sources of cellulose and the extraction process [28].

The researchers of this work used waste jute fiber, an agro-waste, as the choice of starting material because of its excellent characteristics, such as biodegradability, low cost, low density, nontoxicity, and biocompatibility [29,30]. Jute bast fiber is made from the inner bast tissue of the plant’s stem bark of *Corchorus olitorius* (Tossa jute) and *Corchorus capsularis* (white jute). The former is used in micro- and nano-research and commercial applications to obtain cellulose. Jute stem contains a high volume of cellulose (63% for capsularis and 59% for olitorius) that can be harvested in 4–6 months, saving the forest, and meeting the world’s cellulose and wood needs [29,30,31].

According to the literature, waste jute fiber is very rarely used as the primary CNC source. In this study, we produced cellulose nano-whiskers from waste jute fibers using the most efficient method of sulfuric acid hydrolysis, followed by the application of a new technique for the extraction of CNC using a low-density organic solvent through centrifugation, which took significantly less time than the usual membrane dialysis method, which typically requires 4–5 days [32]. In contrast to employing organic solvents, in this instance, cellulose extraction was also carried out using low-concentration alkali pre-treatment, followed by high-concentration alkali treatment at a high temperature with anthraquinone for cellulose pulping.

## 2. Materials and Method

### 2.1. Raw Materials’ Collection

This study used waste jute fibers collected from Shamsher Jute Mill Ltd., Narshingdi, Bangladesh. Visible dirty materials were removed from the fibers, followed by manually mincing to prepare the sample for processing.

### 2.2. Extraction of Pure Cellulose

#### 2.2.1. Pretreatment of Raw Jute Fibers

Minced waste jute fibers were dried at 90 °C and chopped into little pieces (1–2 cm). These jute fibers were soaked in 1 wt% NaOH solution (solid:liquid = 1:10) and treated at 150 °C for 2 h in a High-Pressure Autoclave Reactor (Model IV; CarlRoth, Germany) to remove most non-cellulosic contents. After pretreatment, reacted jute fibers were removed from the reactor and rinsed with distilled water until red litmus paper showed neutralization. Finally, the washed fiber mass was dried at 60 °C for 24 h.

#### 2.2.2. Alkali Treatment of Jute Fiber with Anthraquinone

To eliminate hemicellulose, lignin, and other non-cellulosic structures from pretreated dry jute fibers, a 20% (*w*/*w*) NaOH and 0.5% anthraquinone mixed solution was used. Dry pretreated fibers were appropriately soaked in the above solution at a solid-to-liquid ratio of 1:20 and placed in the same reactor mentioned above. Delignification was performed at 170 °C for 2 h. Treated fibers were collected from the reactor and washed with distilled water until neutralization. Alkali-extracted pulp was bleached by several steps using ClO_2_ bleaching–NaOH extraction–ClO_2_ bleaching–NaOH extraction–H_2_O_2_ bleaching. These bleached fibers were dried at 60 °C for 24 h.

### 2.3. Synthesis of Cellulose Nanocrystals (CNCs) by Acid Hydrolysis

Cellulose (10 g) was hydrolyzed in sulfuric acid solution (58% to 62%) from 20 to 45 min at 40 °C at a solid-to-liquid ratio of 1:20. Initially, CNC suspension was centrifuged and washed repeatedly at 6000 rpm for 10 min. Additionally, suspended CNCs were then dispersed in 2:1 acetone in water and extracted by centrifuging several times at 8000 rpm for 5 min. Following each extraction, the sediment was collected and reconstituted in distilled water. Then, a probe sonicator (probe #6, start time 1 s, finish time 0.5 s) was used to sonicate the suspension at room temperature for 20 min in order to disperse the crystals into a stable suspension. Cellulose nanocrystal suspensions were dried in an oven drier at 100 °C for 30 h. A mortar and pestle smashed tiny film-like crystals into powder. A nano-sieve separated nanocrystal powder and collected it into vials, weighed, and leveled the CNC powder. The percent yield of nanocrystals was calculated based on the initial cellulose amount.

### 2.4. Characterization of Prepared Cellulose Nanocrystals (CNCs)

#### 2.4.1. Particle Size Distribution (PSDs) and Zeta Potential

With the help of the Malvern Zeta Sizer NanoZS90 (Malvern Instruments Ltd., Worcestershire, UK), the particle size distribution and zeta potential of CNC samples were measured by DLS to produce an average value at the measuring site of 0.4 nm, with the temperature of 25 °C. The average CNC size was also measured using the Zeta Sizer at temperature 25 °C, angle 90° and 173°, and a refractive index of 1.333, using water as a solvent.

#### 2.4.2. Fourier Transform Infrared Spectroscopy (FTIR)

On an IRSpirit, Shimadzu spectrophotometer, the cellulose nanocrystal samples’ FTIR spectra were captured. In transmission mode, a total of 20 cumulative scans were performed at a resolution of 4 cm^−1^ and frequencies between 4000 and 650 cm^−1^.

#### 2.4.3. X-ray Diffraction (XRD)

The crystal structure of the jute cellulose nano-whiskers (150 mg) was investigated using an X-ray diffractometer (D/MAX-1200, Rigaku, Japan), with diffraction angles 2θ ranging from 5 to 80 degrees. To measure the crystallinity of materials, a profile analysis was performed using a peak fitting tool using Gaussian line forms. With the help of a 10 kV X-ray generator (Rigaku RINT2000), the X-ray diffraction pattern was obtained using Kb-filtered Cu kα radiation. Origin 2019 pro software was used to evaluate the crystallinity and crystallite size of each tested sample. The crystallinity index and crystallite size for all samples were determined using the formula described earlier [33,34].

#### 2.4.4. Thermal Analysis

The thermal stability, crystalline nature, and pyrolysis behavior of nanocrystal polymers were measured by different thermal analyses, such as TG (thermogravimetry), DTG (derivative thermogravimetry), and DTA (differential thermal analysis), with the help of the TG/DTA EXTAR 6000 STATION, Seiko Instrument Inc., Chiba, Japan. As a function of temperature, the instrument’s resolution was 0.02 μg. The running of the instrument was carried out at a heating speed of 20 °C min^−1^ from ambient temperature to 600 °C, with high-purity nitrogen flow at a rate of 50 mL·min^−1.^

#### 2.4.5. Atomic Force Microscopy (AFM)

The morphology and topography of the CNCs were evaluated with the help of AFM (Easyscan2 FlexAFM, Nanosurf, Liestal, Switzerland). A drop of the aqueous suspension of CNCs was first dried on a glass slide for imaging. The scans were then obtained in semi-contact mode in the air.

## 3. Results and Discussion

### 3.1. Particle Size and Particle Size Distribution (PSDs) of Prepared Samples

Nanocellulose can be prepared by hydrolyzing cellulose with acid (sulfuric acid, hydrochloric acid, nitric acid, phosphoric acid), oxidation by TEMPO, the mechanical method, the biological enzyme method, and the steam explosion method [5,10,19]. The acid hydrolysis method is simple, and at the same time, the acid could be recovered. Its degraded sugar by-products can be used for fermentation to produce biofuel. Therefore, acid hydrolysis is still the main method for the rapid preparation of nanocellulose. While using sulfuric acid to prepare CNC, sulfate groups are attached to the surface of cellulose nanocrystals. This results in electrostatic repulsion among the CNCs, and thus stabilizes aqueous CNC suspensions. In addition to sulfuric acid, other acids such as hydrochloric acid and phosphoric acid can also be used to prepare nanocellulose, but their preference is less than that of sulfuric acid due to the production of less stable suspensions caused by the absence of charges at the CNCs’ surface [10]. In the present study, we have used anthraquinone (AQ) as an oxidant in the soda pulping process of waste jute fibers to remove lignin from the jute fibers. It acts as a catalyst for redox reactions, which occur during the pulping process for the removal of lignin. AQ helps to increase the rate of removal of lignin, decrease the degradation of carbohydrate, increase the pulp yield, and enable the process with reduced time [12].

From the investigation, it was found that the cellulose nano-whiskers’ particle size length was around 800 nm, with some variance. Kasyapi reported nano-whiskers from jute with a slightly different approach, approximately 550 nm, which is lower than this experiment [33]. In contrast, other sources, such as tunicates and valonia, produced cellulose nanocrystals greater than 1000 nm [35,36]. Table 1 demonstrates that a high reaction time with a comparatively high acid concentration yielded significantly smaller nanocrystals. Cellulose is a polysaccharide composed of D-glucopyranose linked by the 1,4-β glycoside bond. The hydroxyl groups in each glucose unit form a strong hydrogen bond with the adjacent glucose unit in the same chain and with the different chain with the formation of a strong ordered hydrogen bond network, called the crystalline region and the amorphous region, corresponding to parts where the bonds are broken, and the ordered arrangement is lost [3]. Strong acid treatment of the cellulose causes it to hydrolyze the amorphous region, keeping the crystalline region unaffected. Treatment with a high concentration of acid for a long time completely degrades the amorphous region, which might be responsible for the smaller grain length of CNCs [10]. The percent yield value of the prepared CNCs also decreases due to the increased concentration of sulfuric acid and the treatment time.

**Figure 1:**
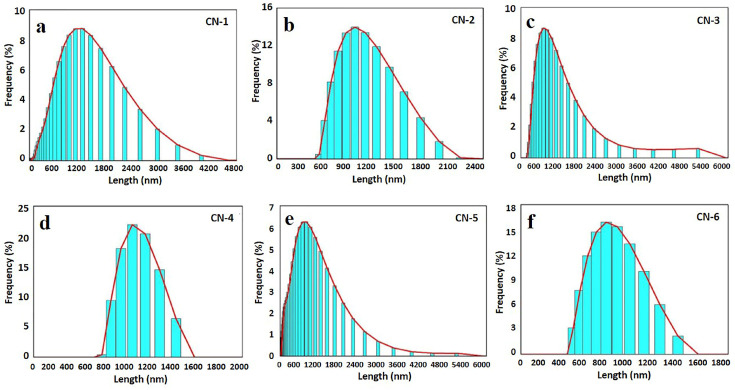
Particle size distribution (by DLS method) of prepared CNC suspensions of (**a**) CN-1, (**b**) CN-2, (**c**) CN-3, (**d**) CN-4, (**e**) CN-5, and (**f**) CN-6.

Cellulose nanocrystal size distribution is shown in Figure 1, in which CN-1, CN-3, and CN-5 are positively skewed distribution curves, whereas CN-2, CN-4, and CN-6 infer standard distribution curves. It implies that a shorter reaction time produces nanocrystals with a higher range toward comparatively higher length sizes; on the other hand, a longer reaction time produces a smaller range of nanocrystals with shorter length sizes. Moreover, these size distribution curves manifest a slight increase in the acid concentration, giving rise to shorter lengths of cellulose nanocrystals. Other researchers have similarly documented the impact of the reaction temperature and acid concentration on the CNC length [37].

### 3.2. Zeta Potential of CNC Suspensions

Zeta potential is an important factor for comprehending the stability of the CNC suspensions that have been manufactured. Zeta potential characterizes the electrostatic repulsion of the particles due to the existence of charge on the CNC particles. This electrostatic repulsion constrains the particles from combining with each other and thus explains the stability of CNC particles in an aqueous suspension [38]. The average values of the zeta potential of different samples are listed in Table 1.

**Figure 2:**
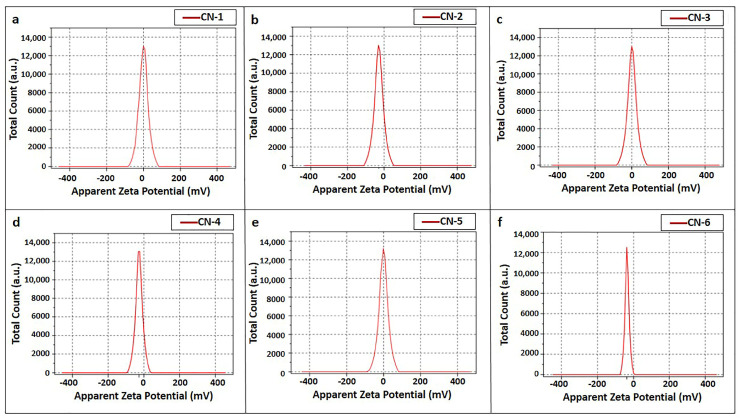
Zeta potential curves from the CNC suspension sample by the Nano Zeta Sizer: (**a**) CN-1, (**b**) CN-2, (**c**) CN-3, (**d**) CN-4, (**e**) CN-5, and (**f**) CN-6.

**Figure 3:**
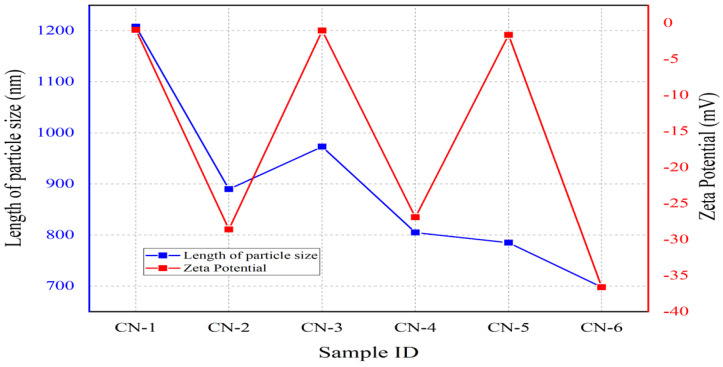
Nanocrystals’ length size and zeta potential correlation.

Zeta potential frequency distribution curves are sketched in Figure 2 and depict the average zeta potential values of the prepared nano-whiskers’ suspensions. A noteworthy change in zeta potential values took place in terms of the reaction time: nanocrystals produced with a higher reaction time (45 min) exhibited a greater surface charge, in the range of around −28 mV to −37 mV, in comparison with a lower reaction time (20 min), which developed a very minor level of surface charge of about −0.9 mV to −1.61 mV. A higher reaction time enhanced the attachment of SO_4_^2−^ ions on the surface of immediately produced nanocrystals, resulting in a greater surface negative potential. Cellulose nanocrystal suspensions are considered stable when the zeta potential value is more than 30 mV, whereas absolute values less than 15 mV are considered responsible for particle agglomeration; hence, CN-1, CN-3, and CN-5 showed larger particle sizes [38,39]. Figure 3 displays an inference that smaller nanocrystals produced for CN-2, CN-4, and CN-6, due to less agglomeration because of higher zeta potential values with a higher reaction time and concentration, attained more stability.

### 3.3. FTIR Analysis of the CNC Samples

**Figure 4:**
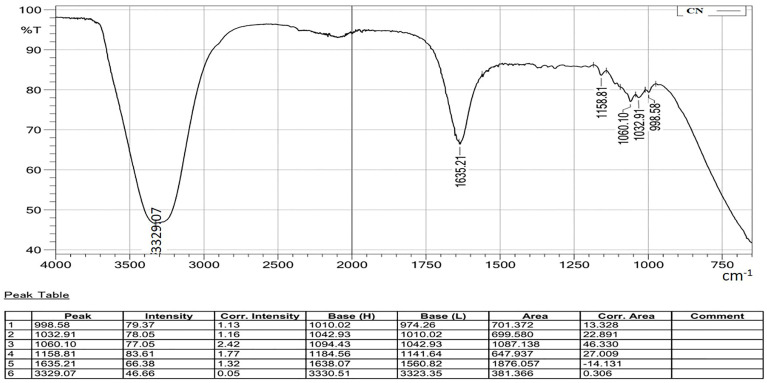
A detailed FTIR spectrum of CN-3, representing the absorption spectra of different groups.

**Figure 5:**
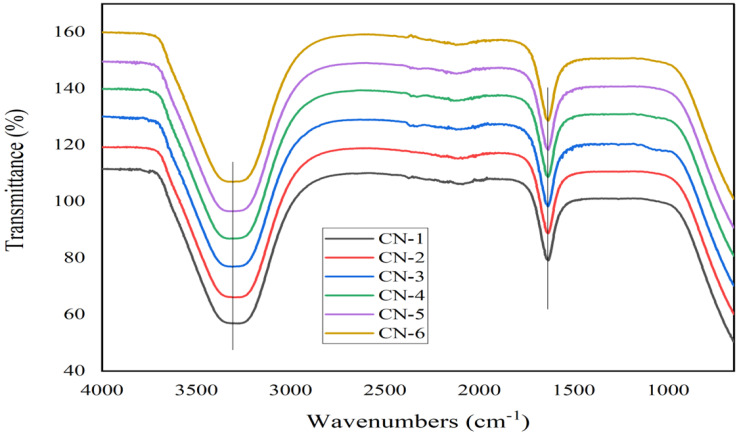
Comparative representation of cellulose nanocrystals’ FTIR spectra of CN-1, CN-2, CN-3, CN-4, CN-5, and CN-6.

The FTIR spectrum confirmed the cellulosic chemical structure of the analyzed nanocrystal suspension. A detailed FTIR spectrum is presented in Figure 4, and comparative spectra are drawn in Figure 5.

All the spectra of the sample exhibited O-H stretching absorption around 3200~3450 cm^−1^, along with C-O stretching absorptions around 1058 and 1112 cm^−1^ [40,41]. The absorption peak at 1158 cm^−1^ indicates C-O-C stretching of the glycosidic ring [39]. The sharp peak at 1635–1640 cm^−1^ shows evidence of absorbed water [42]. The absence of absorption peaks at 1740 cm^−1^ and 1595 cm^−1^ indicates the absence of lignin and hemicellulose in the prepared CNC samples [33].

### 3.4. X-ray Diffraction (XRD) Analysis

The crystallinity index and crystallite size for all samples were calculated using the mentioned formula from the X-ray diffraction curves. The crystallinity index, crystallite size, and 2θ for all samples are summarized in Table 2.

The relative proportion of crystalline material in cellulose has been described using a metric known as the crystallinity index (CrI). Most of the time, the size of a crystallite is the same as the coherent volume in the material for each diffraction peak. It can also mean the size of the grains in a sample of powder.

**Figure 6:**
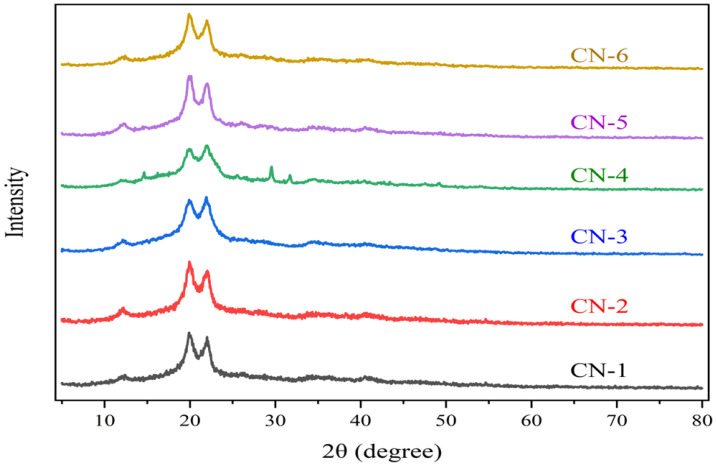
XRD patterns of jute cellulose nanocrystals of CN-1, CN-2, CN-3, CN-4, CN-5, and CN-6.

All the diffractograms in Figure 6 have peak splitting at 20° and 22°, which usually represent the cellulose II structure. According to various studies, 2θ values for CN-1, CN-2, CN-3, and CN-6 reveal the cellulose II polymorphism, and for CN-4 and CN-5, cellulose I and II polymorphic structures [37,43]. The crystallinity index for synthesized CNCs was increased with a longer reaction time as well as a higher acid concentration. High-concentration acid hydrolysis significantly increased the crystallite sizes, i.e., 4.56 nm and 4.27 nm, which were approximately close to cellulose nano-whiskers obtained from rice straw [33,37,44]. A greater crystallinity index (>80%) favors the formation of stable and highly crystalline cellulose nano-whiskers from jute fiber [33].

### 3.5. Thermogravimetric (TGA) Analysis

**Figure 7:**
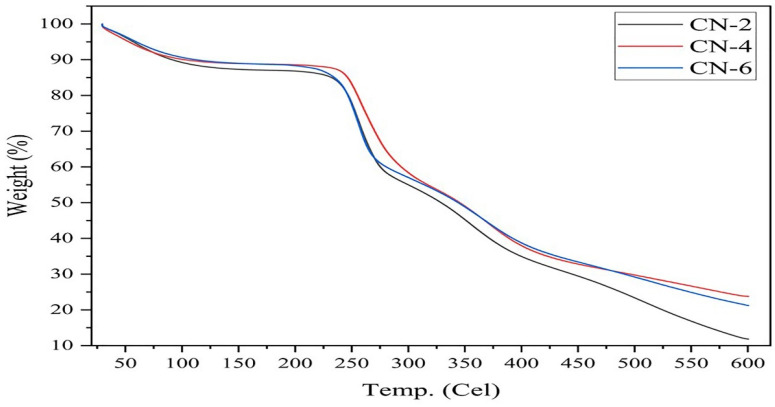
Thermogravimetric (TG) curve of jute CNC samples of CN-2, CN-4, and CN-6.

**Figure 8:**
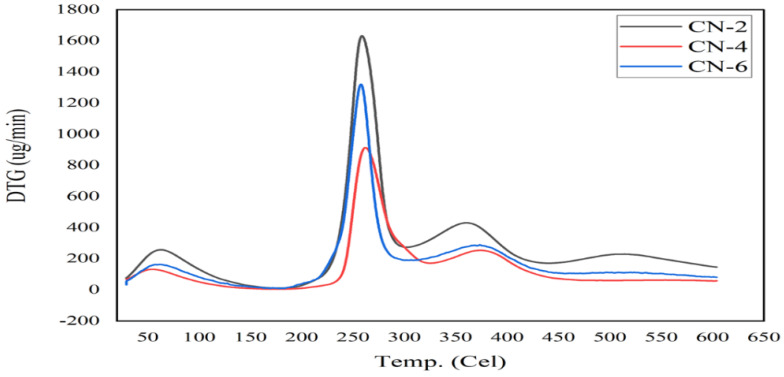
Thermal analysis: DTG curve of jute CNC samples for CN-2, CN-4, and CN-6.

Thermal decomposition, an important characteristic of the stability of nanocrystals for their application, was determined from thermogravimetric studies under nitrogen atmospheres, such as TG and DTG data illustrated in Figure 7 and Figure 8.

From TG (thermogravimetry) and DTG (derivative thermogravimetry) curves, the onset of degradation temperature (Ti) and the maximum degradation temperature (T_max_) were obtained and listed in Table 3.

DTG is the derivative curve of TG, where DTG introduced three degradation peaks at about 63 °C, 258 °C, and 365 °C for samples CN-2, CN-4, and CN-6. Between 250 °C and 350 °C, the weight loss of the samples was caused by the degradation of cellulose due to decomposition of glycosyl units with the formation of burnt residue. The weight loss of the samples at the end of the TGA run could result from the breakdown and oxidation of the burnt residue into volatile products with lower molecular weights [45]. The first degradation peak indicated the evaporation of water molecules from the sample [46,47]. The second peak was seen between 230 °C and 280 °C, while the third peak was seen between 350 °C and 390 °C. These two peaks signify a two-step process of cellulose breakdown [33]. Noticeably, the third peak at around 365 °C confirmed the stable structure of cellulose crystals, but the lower degradation rate implies modification of the surface of the crystals. Major degradation of the amorphous region of CNCs at low-temperature phase (230–280 °C) occurred due to more sulfation and heat accessioned; furthermore, the third degradation at high temperature (350–390 °C) due to less or non-sulfated CNCs. Vanderfleet and other researchers support the mentioned modification by the attachment of sulfate (SO_4_^2−^) ions, due to sulfuric acid hydrolysis, on the surface of the crystals, which undermines the thermal stability maximum degradation observed at 258 °C [48,49,50,51]. Attachment of SO_4_^2−^ ions was evidenced by the zeta potential values (around −28 mV to −36 mV) of CN-2, CN-4, and CN-6. In conclusion, cellulose nanocrystals were well-synthesized and performed thermal degradation at 365 °C.

### 3.6. Differential Thermal Analysis (DTA)

**Figure 9:**
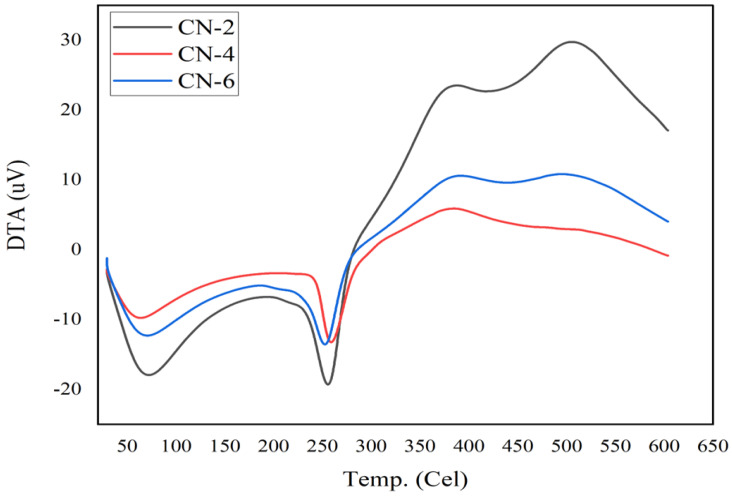
Thermal analysis: DTA curve of jute CNC samples for CN-2, CN-4, and CN-6.

Thermal stability and the nature of prepared crystals were also ascertained by the DTA study, which highlighted the crystal’s prospects in an endothermic or exothermic manner. In Figure 9, the DTA study showed three distinct peaks, where the first two are endothermic and the third one is an exothermic peak.

The temperature and heat flow (μV) of the DTA study of CN-2, CN-4, and CN-6 samples are outlined in Table 4.

Due to moisture loss and evaporation, the first endotherm occurred at temperatures (around 71 °C) significantly lower than 100 °C [37]. The second endotherm, where crystals were degraded at about 258 °C and heat flow was highest, is characteristic of the melting or fusion of crystallites, which revealed the process of decomposition, which was substantiated by TG/DTG analysis. Major decomposition took place at relatively low temperatures as a consequence of a longer acid hydrolysis time and more attachment of sulfate ions on the crystal surface, for which crystals became thermally less stable [28]. The third peak, which is exothermic, denoted the crystallization process of cellulose II crystal, which is thermally more stable than cellulose I crystal. DTA, along with TG/DTG, certified the formation and validated the thermal stability of cellulose nanocrystals from jute.

### 3.7. Atomic Force Microscopy (AFM) Analysis

**Figure 10:**
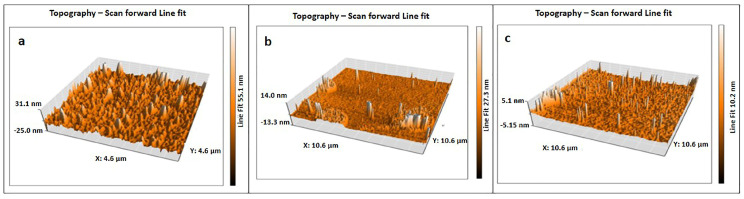
Morphology analysis of jute cellulose nanocrystal samples of (**a**) CN-2, (**b**) CN-4, and (**c**) CN-6 by AFM (3D) images.

The morphology of the nanocrystals is easily understood by AFM analysis as shown in the Figure 10a–c. The AFM photograph revealed rod-like as well as aggregates of CNC particles.

**Figure 11:**
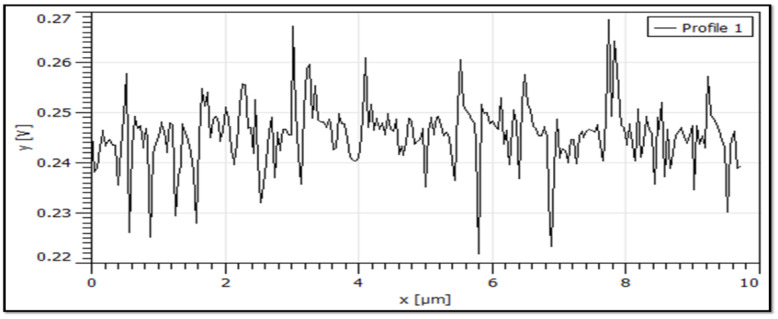
Line scan (small portion) of AFM data of the CN-6 sample to determine the diameter of the nanocrystals.

**Figure 12:**
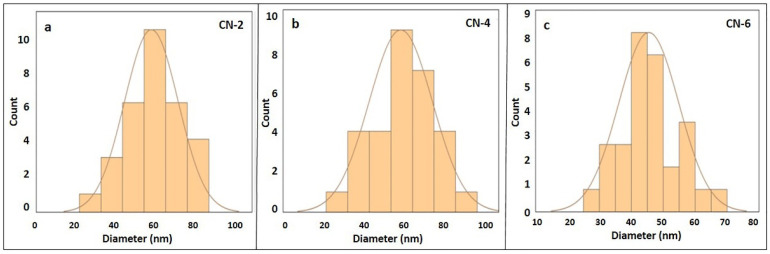
Nanocrystals’ diameter distribution curves of (**a**) CN-2, (**b**) CN-4, and (**c**) CN-6 samples.

Nanoparticles aggregation was reported by few researchers [33,52]. In the present study, clear aggregation of CNC particles is visible in Figure 10a,b, but single crystals are also present in the sample as shown in Figure 10c AFM images. Aggregation of CNC particles are addressed by particle size, zeta potential, and thermal analysis. From AFM 3D images, it is clearly seen that the prepared nanocrystals are rod-like in shape. The length of the crystals seen in the AFM images was evidenced by the DLS method, which showed a minimum of 698 nm and a maximum of 1208 nm. The diameter of the crystals was determined from a line scan of the raw AFM data [44,53]. The diameter of the smaller particle size of CN-2, CN-4, and CN-6 was found at 62 nm, 65 nm, and 45 nm, respectively, via line scan, as shown in Figure 11 and Figure 12. This result is favored by other studies [44], where the diameter was around 50 nm; hence, signifying that an increased acid concentration with reaction time decreased the crystals’ diameter.

## 4. Conclusions

Cellulose nanocrystals (CNCs) were extracted from waste jute fiber by acid hydrolysis. Cellulose extraction was successfully carried out by low-concentration alkali treatment at high temperature and delignification was performed using chlorine dioxide (ClO_2_), sodium hydroxide, and hydrogen peroxide (H_2_O_2_). Acid hydrolysis was conducted for 20 min and 45 min with 58%, 60%, and 62% sulfuric acid concentrations. These variations in parameters produced rod-shaped cellulose nanocrystals. These nanocrystals showed a high degree of crystallinity (above 90%) with a length of 800 ± 100 nm and a diameter of 55 ± 10 nm. As a potential candidate of cellulose nanocrystals, jute fiber produced nano-whiskers with an aspect ratio of above 14. These were characterized by using different techniques, such as Fourier transform infrared spectroscopy (FTIR), X-ray diffraction (XRD), thermogravimetry (TGA), differential thermal analysis (DTA), Nano Zeta Sizer (DLS), and atomic force microscopy (AFM). Therefore, waste jute fiber can be considered a promising source of cellulose nanocrystals for further bio-nanocomposite applications.

## Figures and Tables

**Table 1:** The particle size and zeta potential values of different samples of CNCs.

Sample ID	Reaction Time	Concentration of H_2_SO_4_ (%)	Length of Particle Size	Zeta Potential	% Yield
CN-1	20 min	58% (17.431 N)	1208 nm	−0.912 mV	63.47
CN-2	45 min		890 nm	−28.6 mV	57.29
CN-3	20 min	60% (18.294 N)	973 nm	−1.02 mV	60.16
CN-4	45 min		805 nm	−26.9 mV	51.49
CN-5	20 min	62% (19.178 N)	785 nm	−1.61 mV	54.23
CN-6	45 min		698 nm	−36.6 mV	41.16

**Table 2:** Crystallinity index and crystallite size of different CNC samples.

Sample ID	2θ (Degree)	Crystallinity Index	Crystallite Size
CN-1	12.23, 20.03, 22.01	80.68%	3.052 nm
CN-2	12.21, 19.97, 21.96	90.24%	3.765 nm
CN-3	12.07, 19.93, 21.92	79.73%	2.705 nm
CN-4	11.89, 19.82, 21.96, 29.55, 31.69, 34.4	84.35%	3.765 nm
CN-5	12.14, 17.8, 19.91, 21.97, 35.2, 40.64	89.4%	4.560 nm
CN-6	11.91, 20.01, 22.04	90.91%	4.270 nm

**Table 3:** Different thermal parameters for samples from TGA and DTG curves.

Sample	Ti (°C)	T_max_ (°C)	Residue (wt%)	DTG (mg/min) at T_max_
CN-2	202.1	258.6	12.0	1.632
CN-4	197.6	261.9	23.7	0.914
CN-6	192.4	257.7	21.6	1.309

**Table 4:** DTA parameters of CNC samples.

Sample ID	Reaction Time	% Concentration of H_2_SO_4_	Temp °C	DTA uV
CN-2	45 min	58% (17.431 N)	71.08668518	−17.93228135
			255.9306183	−19.27960668
			419.8621521	22.70405841
CN-4	45 min	60% (18.294 N)	65.72425079	−9.754850507
			259.4009399	−13.23660767
CN-6	45 min	62% (19.178 N)	71.50741577	−12.28114557
			253.3040009	−13.54624414
			435.6995239	9.605033398

## Data Availability

The datasets used and analyzed during the current study are available from the corresponding author upon reasonable request.
